# Antiallergic Effect of the Alpha-Cyclodextrin Moringin Complex in Rat Basophilic Leukaemia (RBL-2H3) Cell Line

**DOI:** 10.1155/2024/8885068

**Published:** 2024-07-25

**Authors:** Ebtisam Yousef A. Alnakeeb, Ahmad Faizal Abdull Razis, Kim Wei Chan, Chau Ling Tham, Yee Han Chan, Anwar Salm Kalifa Kafo, Nuzul Noorahya Jambari, Patrick Rollin, Florence Djedaini-Pilard

**Affiliations:** ^1^ Natural Medicines and Products Research Laboratory Institute of Bioscience Universiti Putra Malaysia, UPM Serdang 43400, Selangor, Malaysia; ^2^ Laboratory of Food Safety and Food Integrity Institute of Tropical Agriculture and Food Security Universiti Putra Malaysia, UPM Serdang 43400, Selangor, Malaysia; ^3^ Department of Food Science Faculty of Food Science and Technology Universiti Putra Malaysia, UPM Serdang 43400, Selangor, Malaysia; ^4^ Department of Biomedical Sciences Faculty of Medicine and Health Sciences Universiti Putra Malaysia, UPM Serdang 43400, Selangor, Malaysia; ^5^ Department of Pathology Faculty of Medicine and Health Sciences Universiti Putra Malaysia, UPM Serdang 43400, Selangor, Malaysia; ^6^ Université d'Orléans et CNRS ICOA, UMR 7311, BP 6759, CEDEX 02, Orléans F-45067, France; ^7^ LG2A UR 7378 Université de Picardie Jules Verne 33 rue Saint Leu—UFR des Sciences, Amiens F-80000, France

## Abstract

Allergic diseases (ADs) are a major concern when it comes to public well-being. *Moringa oleifera* Lam is a tropical plant that is used in traditional medicine due to the presence of isothiocyanate. The present study investigated the antiallergic properties of 4-(*α*-L-rhamnopyranosyloxy)-benzyl isothiocyanate or moringin isolated from *Moringa oleifera* seeds in the form of alpha-cyclodextrin-moringin (*α*-CD/MG) complex on rat basophilic leukaemia (RBL-2H3) cell line at both the early and late stages of an allergic reaction. The *α*-CD/MG complex was initially elucidated using nuclear magnetic resonance (NMR) followed by the 3-(4,5-dimethylthiazol-2-yl)-5-(3-carboxymethoxyphenyl)-2-(4-sulfophenyl)-2H-tetrazolium inner salt proliferation assay to evaluate the cytotoxicity and cell viability with respect to ketotifen fumarate (KF) and *α*-CD/MG. The release of beta-hexosaminidase (*β*-hexosaminidase) and histamine was used to determine the level of inhibition in the early stage while the suppression of the release of prostaglandin (PGD2), tumour necrosis factor-alpha (TNF-*α*), and interleukin (IL-4) was considered in the late stage. Higher concentrations of *α*-CD/MG (5 *μ*M, *p* < 0.001) in mast cell degranulation significantly inhibited the expression of *β*-hexosaminidase, histamine, TNF-*α*, PGD2, and IL-4 in both the early and late stages. Thus, *α*-CD/MG can potentially be developed as an antiallergic drug as it has the ability to inhibit allergic responses in the late and early stages.

## 1. Introduction

The prevalence of common allergic diseases (ADs) such as allergic rhinitis, eczema, asthma, and food allergies has notably increased [1]. An AD refers to any unwanted hypersensitive reactions towards an antigen, which stimulates the body's immunological system [2, 3]. The prevalence of allergy illnesses is global, affects all age groups, and has consistently increased in the past two centuries [4]. Outdoor and indoor allergens, increasing ambient temperatures, air pollution, and early spring with higher levels of airborne pollen contribute to the progression of ADs [5, 6]. These ADs cause numerous problems, particularly in children. Allergies can broadly be classified into two types: early-phase and late-phase reactions. Allergic reactions normally begin one to two minutes post-exposure to an allergen [7]. In the early stage of the reaction, a mast cell degranulation reaction leads to the release of histamine and other granule proteins in the environment, while in the later phase of the reaction, the activated mast cells cause cytokines, leukotrienes, and prostaglandins to be released [8, 9]. In ADs, allergens bind to immunoglobulin E (IgE) and high affinity mast cell receptors. Antigens aid in crosslinking IgE-bound Fc epsilon receptors (FcRI) to initiate the degranulation of mast cells. Most allergic reactions are caused by the interaction between IgE and FcRI receptors [10–13].

Food allergies are considered a significantly severe medical condition. Every year, almost 5% of adults and ∼8% of children in developing countries especially experience food allergies, mainly due to immunoglobulin (Ig) E4, which leads to asthma, nausea, diarrhoea, allergic rhinitis, peptic ulcer, vomiting, allergic dermatitis, allergic shock, and sometimes death [14]. The number of incidences continues to grow each year. Currently, no approved medications exist to completely cure ADs. Medications such as (a) mast cell stabilisers, namely, disodium chromoglycate or ketotifen, (b) antihistamines such as chlorpheniramine maleate, terfenadine, and diphenhydramine, and (c) immune suppressors such as dexamethasone, adrenocortical hormones, and hydrocortisone can be used to relieve allergic symptoms to some extent. However, most of those drugs have undesirable side effects and are also associated with the early recurrence of symptoms. A viable antiallergic strategy would be to develop food-derived antiallergic components, thereby reducing recurrence and side effects. Natural foods contain biologically active compounds such as polyphenols and flavonoids that possess antioxidant and anti-inflammatory characteristics. They have also been found to potentially decrease allergic symptoms [15].

Phytochemicals are currently used to treat and ameliorate the symptoms of ADs. High amounts of sulforaphane, an isothiocyanate, are present in many cruciferous vegetables. Sulforaphane was shown to play a key role in the inhibition of an allergic inflammatory response. It has been found to inhibit the activation of caspase-1 as well as the mitogen-activated protein kinase (MAPK) and nuclear factor of kappa-light-chain-enhancer of activated B cells (NF-KB) signalling pathways, thereby decreasing the levels of inflammatory cytokine [16]. Sulforaphane also decreased the bacterial lipopolysaccharide (LPS)-mediated expression of interleukin-1*β* (IL-1*β*), cyclooxygenase-2 (COX-2), and tumour necrosis factor-alpha (TNF-*α*) as well as the inducible nitric oxide synthase (iNOS) in macrophages [17–19]. Phenethyl isothiocyanate has been found to have a pharmacological impact on inflammatory responses with regard to thymic stromal lymphopoietin (TSLP)-stimulated mast cells [20]. It also substantially decreased interleukin-13 (IL-13) and TNF-*α* levels in human mast cell-1 (HMC-1) and increased TSLP levels. 6-(Methylsulfinyl) hexyl isothiocyanate has been found to decrease COX-2 and cytokine levels as well as inhibited inducible nitric oxide synthase (iNOS), all of which are regarded as key mediators of inflammatory responses. These findings emphasised the potential of phytochemicals derived of glucosinolates in the management of inflammatory reactions in mast cells and allergic diseases [21].

*M. oleifera* plant holds various bioactive substances such as flavonoids, polyphenols, carotenoids, and ascorbic acid, with the highest concentration of polyphenols found in its leaves. Its leaves also contain other phytochemicals including glucosinolates, rhamnose, and glucose. The flavonoids present in *M. oleifera* have been found to safeguard against oxidative stress-induced chronic illnesses such as cardiovascular disorders and cancer [22]. Moringin (MG) is the isothiocyanate resulting from the enzymatic hydrolysis of glucomoringin, the major glucosinolate present in the plant [23–30]. It was reported to suppress lipopolysaccharide-induced inflammation in RAW 264.7 macrophage cells [31]. Multiple studies have reported methods for glucomoringin isolation from *M. oleifera* L. seeds. Giacoppo et al. [25] were the first to incorporate MG into alpha-cyclodextrin (a-CD) which was found to be effective at stabilising and solubilising MG [26], thus eliciting and improving its bioactivity.

The antiallergic effect of *α*-CD/MG could be also linked to its suppressive effect on the degranulation of mast cells, which play a key role in the inflammatory process. Once activated, a mast cell can either release mediators or compounds that induce inflammation. Thus, this study assessed the antiallergic impact of *α*-CD/MG on rat basophilic leukaemia (RBL-2H3) cells that had been stimulated with dinitrophenol-bovine serum albumin (DNP-BSA) and sensitised using dinitrophenyl immunoglobulin E (DNP-IgE).

## 2. Materials and Methods

### 2.1. *α*-CD/MG Complex

As part of the Universiti Putra Malaysia (UPM)/France Hubert Curien Partnerships (PHC) Hibiscus-2020 collaboration, freeze-dried *α*-CD/MG inclusion complex was prepared as already described [26] and provided by LG2A (Université de Picardie Jules Verne, France). Briefly, moringin was isolated from *M. oleifera* seeds (cake powder PKM2 supplied by Indena India Pvt. Ltd.; Bangalore, India) using modified methods; based on the molecular weights and a 1 : 1 M ratio of the two constituents, a soluble complex was acquired by adding 103 mg of solid moringin to a solution of 300 mg *α*-CD (Wacker Chemie AG, Germany) in 3.0 mL of water. The mixture was filtered with 0.45 *μ*m filter, then freeze-dried (Edwards model DO1; Milan, Italy) and characterised using NMR. The *α*-CD/MG was then kept at room temperature before use.

### 2.2. Characterisation of the *α*-CD/MG Complex Using NMR

To characterise the complexation between *α*-CD and MG, experiments with 1D and 2D NMR were conducted at 300 K using a 500 MHz Varian NMR machine. Employing trimethylsilypropanoic (TSP) as the internal standard, MG and freeze-dried *α*-CD were reconstituted in deuterium oxide (D_2_O) until a concentration of 5 mM was obtained. The MestReNova software was then used to analyse the hydrogen-1 nuclear magnetic resonance (^1^H-NMR) experiments [27].

### 2.3. RBL-2H3 Cell Culture and Maintenance

The RBL-2H3 cell line was supplied by the American Type Culture Collection (ATCC); the cell line was grown in minimum essential media (MEM) medium and sodium pyruvate containing 10% foetal bovine serum. A subculture of RBL-2H3 cells was then placed on fresh T25 tissue culture flasks. This procedure was applied after the cells had been incubated at 37°C in 5% CO_2_ humidified incubators. Additionally, the cytotoxicity assay of various *α*-CD/MG concentrations (1.25, 2.5, 5, and 10 *μ*Μ) on RBL-2H3 was performed as described [25, 28]. Ketotifen fumarate (KF) was chosen as positive control in this study with a concentration of 75 *μ*M.

### 2.4. RBL-2H3 Cell Viability Measurement Using MTS Assay

The cells were grown in 96-well plates at a density of 2 × 10^4^ cells/well, using an enriched medium (100 *μ*L) from the ATCC. Before reconstituting the cells with a test sample at 100 *μ*L, they were incubated for 24 hours at a temperature of 37°C inside a humidified incubator with 5% CO_2_ and 95% air. After incubation with 3-(4,5-dimethylthiazol-2-yl)-5-(3-carboxymethoxyphenyl)-2-(4-sulfophenyl)-2H-tetrazolium inner salt (MTS) reagent at 317 *μ*g/ml for 4 hours, absorbance was read at 490 nm using Synergy H1 absorbance multimode microplate reader (BioTek, Winosski, VT, USA). The results at 24, 48, and 72 hours were expressed as cell viability percentages which were calculated using the following formula, where OD refers to the optical density [29]:

(1) $$\begin{array}{c} p\text{ercentage of cell viability}=\frac{{\text{OD}}_{490}\;\left(\text{sample}\right)-{\text{OD}}_{490}\;\left(\text{blank}\right)}{{\text{OD}}_{490}\;\left(\text{control}\right)-{\text{OD}}_{490}\;\left(\text{blank}\right)}*100. \end{array}$$

### 2.5. Histamine Release Measurement

A study was carried out to evaluate the impact of *α*-CD/MG on the release of preformed mediators during mast cell degranulation in relation to the release of histamine. A 2 × 10^5^ cells were seeded in a 6-well plate for 24 hours and then sensitised with DNP-IgE at 0.1 *μ*g/mL for 24 hours. The sensitised cells were treated for 24 hours with *α*-CD/MG at concentrations of 0.625, 1.25, 2.5, and 5 *μ*M after being washed with phosphate buffer saline. The pretreated cells were then treated with DNP-BSA for 6 hours followed by the collection of the culture media and centrifuged for 20 minutes at 4°C (1000 × g). The histamine level in the cultured supernatant was analysed using a histamine enzyme linked immunosorbent assay (EIA) kit, with the absorbance measured at 405 nm using a Synergy H1 absorbance multimode microplate reader (BioTek, Winooski, VT, USA) [30].

### 2.6. *β*-Hexosaminidase Release Measurement

*β*-hexosaminidase activity was measured using the previously described technique with some modifications [30]. Each group of supernatant was transferred and centrifuged for 20 minutes at 4°C (1000 × g), and intracellular-hexosaminidase lysing cells were released using 0.1% Triton X-100 solution. This was followed by the preparation of the black 96-well plates, which was accomplished by transferring 50 *μ*L of cell lysate and 50 *μ*L of culture supernatant to the various wells, adding the substrate solution, and then incubating the well plates at 37°C for 15 minutes. The reaction ended with the addition of 100 *μ*L of neutralisation buffer to each well. Microplate fluorometers were then used to measure the excitation wavelength (365 nm) and emission wavelength (450 nm) using a Synergy H1 absorbance multimode microplate reader (BioTek, Winosski, VT, USA). The following formula was then used to determine the degranulation percentage:

(2) $$\begin{array}{c} \%\text{ degranulation}=\frac{\left(\text{OD supernatant}\right)}{\left(\text{OD supernatant}+\text{OD cell lysis}\right)}*100. \end{array}$$

### 2.7. Prostaglandin 2 (PGD2), TNF-*α*, and IL-4 Releases Measurement

The effect of *α*-CD/MG on the release of mediators by RBL-2H3 cells (2 × 10^6^ cells/mL) treated for 24 hours with 1 *μ*g/mL of DNP-IgE was also determined. The cells were washed with phosphate buffer saline and pretreated for 24 hours with *α*-CD/MG (0.625, 1.25, 2.5, and 5 *μ*M). The cells were then treated with DNP-BSA (1 *μ*g/mL) for 24 hours after the pretreatment. The supernatant was centrifuged at 4°C for 20 minutes after being transferred into centrifuge tubes. The released mediators were measured using an ELISA kit, according to the manufacturer's instructions [9].

### 2.8. Statistical Analysis

Data were presented as mean ± standard deviation. A one-way analysis of variance (ANOVA) with Tukey's multiple comparisons was carried out using the Prism Statistical Package version 9.0 to find the difference between the means. A difference of *p* < 0.05 was considered as statistically significant.

## 3. Results and Discussion

### 3.1. NMR Characterisation of the *α*-CD/MG Complex

Trimethylsilylpropanoic acid (TSP) was used as an internal standard in deuterium oxide (D_2_O) to dissolve 5 mM of *α*-CD/MG in a solution. Proton (1D) and total correlation spectroscopy (TOCSY) (2D) nuclear magnetic resonance (NMR) were conducted to characterise the samples. Nuclear Overhauser Effect (NOE) is unique among NMR methods because it depends only on the spatial proximity between protons. In other words, the strength of the NOE gives information on how close two protons are in solution, not only belonging to the same molecule (intramolecular) but also to two different molecules (supramolecular). Taking into account the medium size of the supramolecular complex, the ROESY experiment (rotating frame NOE) was selected in our case.

**Figure 1:**
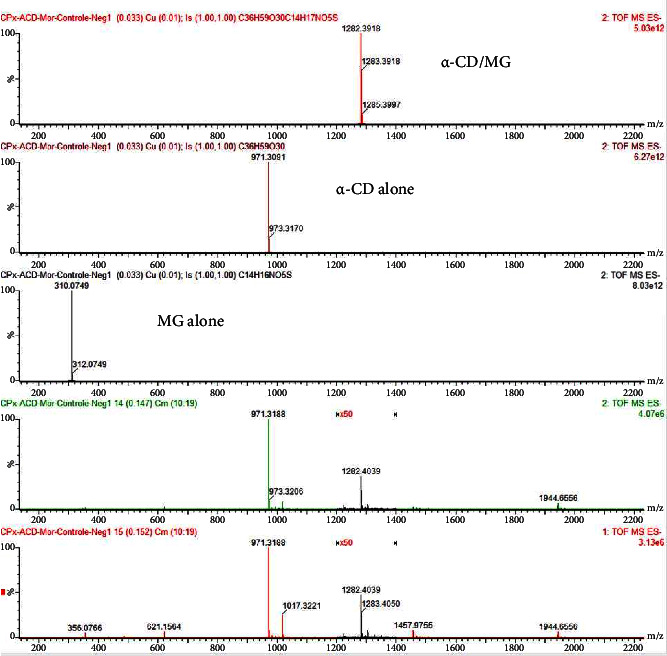
Mass spectrometry of the complex (*α*-CD/MG), *α*-CD, and MG alone.

**Figure 2:**
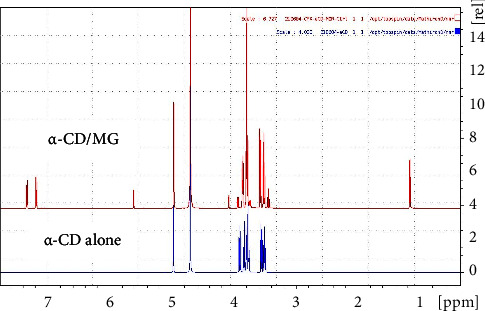
Proton NMR of the complex (*α*-CD/MG) and *α*-CD alone.

**Figure 3:**
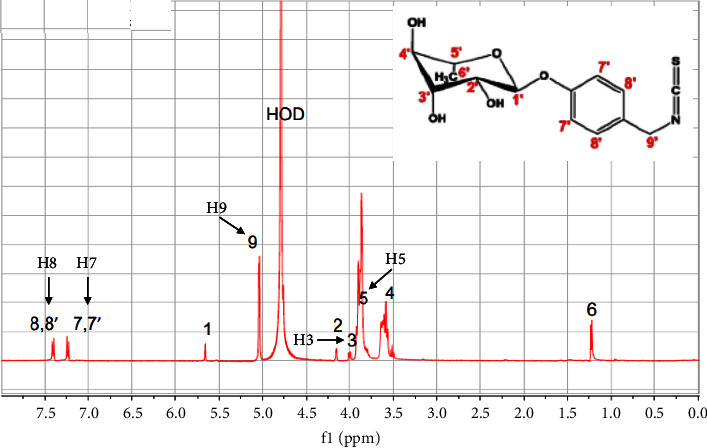
The proton (^1^H)-NMR spectra of *α*-CD/MG (5 mM in D_2_O).

First the *α*CD/MG complex was evidenced by mass spectrometry in water since *α*CD/MG mixture infused in the electrospray source operating in negative ionization mode (ESI-) provides only the expected [*α*-CD/MG-H]- ion at m/z 1282.40 as displayed on Figure 1. Comparison of the ^1^H-NMR spectra of the isolated and complex compounds performed by our French counterpart shows typical variations in the chemical shift of the signals of specific protons (aromatic H7, H8, and H9 of MG and inner H3 and H5 of *α*-CD, Figures 2 and 3), in agreement with the formation of the inclusion complex as displayed in Figure 3. These observations were confirmed by TROSY experiments highlighting the spatial proximities between the same protons of guest and host in agreement with the results already described [26, 27]. Finally, TOCSY experiment was carried out to check the chemical integrity of both species in the inclusion complex.

### 3.2. Effect of *α*-CD/MG on the Viability of RBL-2H3 Cells

**Figure 4:**
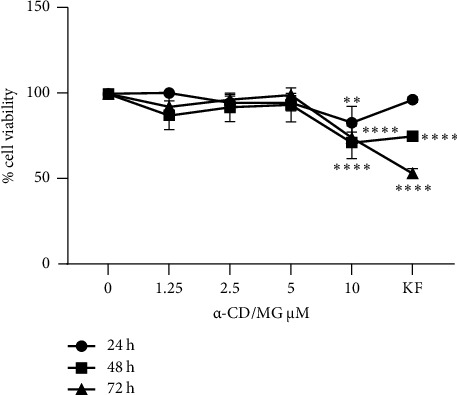
Cytotoxic effect of 1.25, 2.5, 5.0, and 10 *μ*M of *α*-CD/MG on RBL-2H3 cells after (a) 1 day, (b) 2 days, and (c) 3 days of incubation. Values are mean ± SD of three independent experiments. ^*∗∗*^*p* < 0.001 and ^*∗∗∗∗*^*p* <  0.0001 indicating statistically significant in comparison with the untreated control, ketotifen fumarate (KF).

The *α*-CD/MG complex was assessed to determine the appropriate dosage and its impact on the viability of RBL-2H3 cells. The highest concentration (10 *μ*M; *p* < 0.001) of *α*-CD/MG led to a lower RBL-2H3 cell viability (Figure 4). However, cell proliferation increased in an *α*-CD/MG concentration-dependent manner (1.25, 2.5 and 5 *μ*M; *p* > 0.05). *α*-CD/MG also had a more significant impact on the RBL-2H3 cells than ketotifen fumarate (KF) under similar conditions. The concentrations of 1.25, 2.5, and 5 *μ*M were chosen for further experiment because the cell viability is about 80% which is considered non-toxic.

The present study revealed a considerable increase in RBL-2H3 cell proliferation depending on the concentration of *α*-CD/MG used. Therefore, *α*-CD/MG can safely and effectively stimulate cell proliferation. Multiple studies have tested and assessed the toxicity of *Moringa* plant and its derivatives and concluded that they are safe at low concentrations. For instance, concentrations of 7.81, 15.62, and 31.25 *µ*g/mL of *Moringa*-isolated compounds have been examined and confirmed the viability of RBL-2H3 cells under such conditions [30]. *α*-cyclodextrin (*α*-CD) and *α*-CD/MG have also been found to significantly increase the viability of the RAW 264.7 cell line [31]. This is due to the synergistic effect of the complex (*α*-CD/MG) which able to maintain the cells viability until its elicited cell inhibition at the higher concentration. Allergy studies typically use RBL cells *in vitro* models due to their high sensitivity towards IgE and its Fc*ε*RI receptors. Mast cells are a suitable substitute for RBL cells as they are far more stable in tissue culture. Tan et al. [9] and Lorz et al. [29] respectively exposed RBL-2H3 cells to 50 mg/ml of a fraction of *Panax ginseng* (*BIOGF1K*) and 10 to 40 *μ*M of 2,4,6-trihydroxy-3-geranylacetophenone (tHGA) and found them to be non-cytotoxic to the cells [9, 29].

### 3.3. Antihistamine Effect of *α*-CD/MG on RBL-2H3 Cells

**Figure 5:**
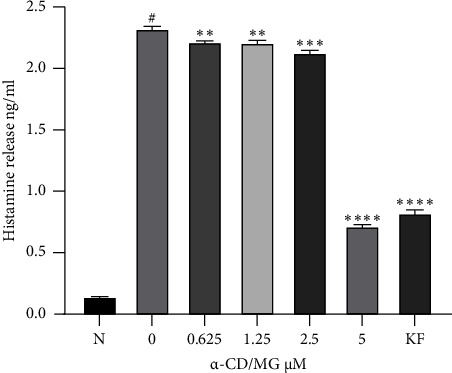
Antihistaminic effects of *α*-CD/MG on RBL-2H3 cells. The RBL-2H3 cells were sensitised with IgE for 24 hours and then stimulated with 1 *μ*g/mL of DNP-BSA for 6 hours pretreatment with 0.625, 1.25, 2.5, and 5.0 *μ*M of *α*-CD/MG. An ELISA was used as per the manufacturer's instructions to measure the histamine levels. The data of the three determinants (*n* = 3) are presented as mean ± SD, ^*∗∗*^*p* < 0.001, ^*∗∗∗*^*p* < 0.0001, and ^*∗∗∗∗*^*p* < 0.0001 indicating statistically significant in comparison with the untreated group. ^#^*p* < 0.05 in comparison with the normal group (N).

At concentrations of 5.0, 2.5, 1.25, and 0.625 *μ*M, the *α*-CD/MG showed a dose-dependent inhibition of histamine release. The *p* value in comparison to the untreated cells was less than 0.001. The level of RBL-2H3 degranulation was determined by measuring the histamine release, as histamine significantly affects the degranulation of mast cells. Thus, a preformed mediator was released due to mast cell activation. As seen in Figure 5, the RBL-2H3 cells were stimulated for six hours with IgE-antigen complexes, which showed a considerably higher release of histamine with DNP-BSA. When pretreated with *α*-CD/MG in a concentration-dependent manner, histamine release decreased leading to a significant reduction in degranulation. More specifically, 5 *μ*M of *α*-CD/MG was found to significantly decrease histamine release in the RBL-2H3 cells that had been stimulated with the IgE-antigen complex (DNP-IgE and DNP-BSA). A concentration of 75 *μ*M of KF was required to elicit the same effect at 5 *μ*M of *α*-CD/MG on the RBL-2H3 cells (positive control).

The inflammatory mediators that cause allergic reactions are released during mast cell degranulation. Among those mediators, histamine protein plays a key role in allergic reactions [32]. The findings of the present study indicated that *α*-CD/MG decreased the release of histamine. The impact of *α*-CD/MG on mast cell degranulation was evaluated to measure the release of the preformed mediator. The results of the histamine assay indicated that *α*-CD/MG suppressed the release of histamine by IgE-antigen complex-stimulated RBL-2H3 cells by attenuating the degranulation process. Other studies have similarly found that ginsenoside Rg3 (G-Rg3) inhibited the release of histamine by stimulated mast cells, whereby 2 to 50 *μ*g/mL of G-Rg3 decreased the activated RBL-2H3 cells in a dose-dependent manner. More specifically, 2 × 10^5^ RBL-2H3 cells per well were pretreated with G-Rg3 for 30 minutes, then stimulated with 10 mg/mL of DNP-BSA for 24 hours and sensitised with 100 ng/mL of DNP-IgE [32].

In order to assess the inhibition of mast cell degranulation, the amount of histamine released from activated RBL-2H3 cells was quantified. This is in line with the investigation aimed to determine whether treatment with new fraction of Korean ginseng containing a high amount of compound K, named *BIOGF1K* [29], berberine [33], curcumin [34], and quercetin [35], could impede the release of histamine. All these compounds have been found to contain lower histamine levels in a concentration-dependent manner.

### 3.4. Inhibition of *β*eta-Hexosaminidase Release by *α*-CD/MG

**Figure 6:**
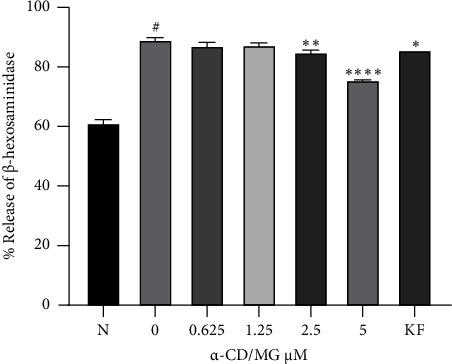
*α*-CD/MG inhibited the release of *β*-hexosaminidase in activated RBL-2H3 cells. The RBL-2H3 cells were sensitised with IgE for 24 hours and then stimulated with 1 *μ*g/mL of DNP-BSA for 6 hours pretreatment with 0.625, 1.25, 2.5, and 5.0 *μ*M of *α*-CD/MG. An ELISA was used as per the manufacturer's instructions to measure the histamine levels. The data of the three determinants (*n* = 3) are presented as mean ± SD, ^*∗*^*p* < 0.05, ^*∗∗*^*p* < 0.001, and ^*∗∗∗∗*^*p* < 0.0001 indicating statistically significant in comparison with the untreated group. ^#^*p* < 0.05 in comparison with the normal group (N).

As seen in Figure 6, an ELISA assay demonstrated that RBL-2H3 cells have released histamine post-sensitisation with DNP-IgE and post-stimulation with DNP-BSA for 24 hours. Furthermore, 5 and 2.5 *μ*M of *α*-CD/MG significantly inhibited the release of *β*-hexosaminidase, with *p* < 0.001 compared to the untreated cells. The extent of RBL-2H3 degranulation was determined by measuring the release of *β*-hexosaminidase, as it plays a primary role in mast cell degranulation. The preformed mediator is released in the early stages of mast cell activation. DNP-BSA increased the release of *β*-hexosaminidase in RBL-2H3 cells that had been activated with IgE-antigen complexes for 6 hours. Pretreated RBL cells with *α*-CD/MG led to a concentration-dependent reduction in RBL cells degranulation, as evidenced by a decrease in the amount of *β*-hexosaminidase released. Concentrations of 5 and 2.5 *μ*M *α*-CD/MG significantly decreased the amount of *β*-hexosaminidase released by RBL-2H3 cells that had been stimulated using an IgE-antigen complex. The *α*-CD/MG had the same effect on the RBL-2H3 cells as 75 *μ*M of KF (positive control).

The present study also found that *α*-CD/MG inhibited the release of *β*-hexosaminidase. The impact of *α*-CD/MG on the degranulation of mast cells was evaluated by ascertaining the release of the preformed mediator. The results of the *β*-hexosaminidase assay indicated that *α*-CD/MG had an inhibitory effect on the degranulation process, as observed by the significant reduction in *β*-hexosaminidase synthesis when an IgE-antigen complex was used to stimulate the RBL-2H3 cells.

Similarly, some studies discovered that *Rubus suavissimus* (Tencha) and black soybean hull extract (BSHE), at concentrations of 125 *μ*g/mL and 500 *μ*g/mL, respectively, decreased the activity of *β*-hexosaminidase [36]. *α*-CD/MG at concentrations of 5.0 and 2.5 *μ*M has decreased the effect of *β*-hexosaminidase on RBL-2H3 cells in a dose-dependent manner. Another study also has found that high concentrations of saponarin (20 and 40 *μ*M) reduced the effect of *β*-hexosaminidase [37].

In other aspects, a concentration of 30 *μ*g/mL of zinc oxide (ZnO) nanoparticles has been found to inhibit the synthesis of *β*-hexosaminidase by RBL-2H3 mast cells [38]. Conversely, a low-dose (0.05 Gy) of ionising radiation, irrespective of the duration, was found to significantly decrease the degree of mast cell degranulation due to mast cell activation (*β*-hexosaminidase). Therefore, low-dose ionising radiation has therapeutic and preventive effects on signalling routes and degranulation. It was hypothesised that low-dose ionising radiation decreased degranulation and inflammatory cytokine expression in activated mast cells and decreased allergy symptoms *in vivo* [39].

### 3.5. Effect of *α*-CD/MG on TNF-*α* Release

**Figure 7:**
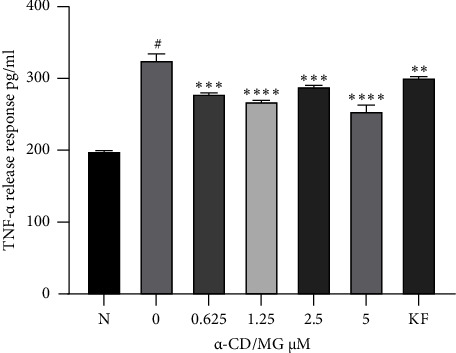
Effect of *α*-CD/MG on TNF-*α* release by activated RBL-2H3 cells. The RBL-2H3 cells were sensitised with IgE for 24 hours and then stimulated with 1 *μ*g/mL of DNP-BSA for 6 hours pretreatment with 0.625, 1.25, 2.5, and 5.0 *μ*M of *α*-CD/MG. An ELISA was used as per the manufacturer's instructions to measure the cytokine concentrations. The data of the two determinants (*n* = 2) are presented as mean ± SD, ^*∗∗*^*p* < 0.001, and ^*∗∗∗*^*p* < 0.0001, and ^*∗∗∗∗*^*p* < 0.0001 indicating statistically significant in comparison with the untreated group. ^#^*p* < 0.05 in comparison with the normal group (N).

An ELISA assay was used to measure the release of TNF-*α* by RBL-2H3 cells sensitised with DNP-IgE and activated with DNP-BSA for 24 hours. The results indicated that 5.0, 2.5, 1.25, and 0.625 *μ*M of *α*-CD/MG significantly inhibited the release of TNF-*α* in a dose-dependent manner (*p* < 0.005) compared to untreated cells (Figure 7). Exposing RBL-2H3 cells to antigen for extended periods has triggered a late-phase interaction, where the amount of the *de novo* pro-inflammatory cytokine TNF-*α* release increased [32].

It was shown that *α*-CD/MG inhibited the release of TNF-*α*. As seen in Figure 5, the IgE-antigen complex-stimulated cells produced significantly higher amounts of TNF-*α* post-24-hour DNP-BSA challenge. However, the amount of TNF-*α* released by the RBL-2H3 cells post-stimulation with the IgE-antigen complex has significantly decreased when treated with 5, 2.5, 1.25, and 0.625 *μ*M of *α*-CD/MG. Furthermore, the amount of *de novo* mediators in the IgE-antigen complex-stimulated cells that had been pretreated with *α*-CD/MG has significantly decreased.

Mast cells are regarded as the only cells that can store the cytokine TNF-*α* in cytoplasmic granules for quick release when activated. Nevertheless, the TNF-*α* released from the cytoplasmic granules of mast cells is considered to follow a different modulation than degranulation [40]. The mast cell stabilisation characteristics of *α*-CD/MG and KF are considered similar. The latter, which was the control drug of this present study, is clinically used as an antiallergen that stabilises mast cells by resisting the malformation of the plasma membrane by degranulating mast cells [9]. Aside from these *de novo* lipid mediators, the antigen responds by activating mast cells that synthesis and release TNF-*α*, which comprises *de novo* pro-inflammatory cytokines that execute vital interactions during the late stages of hypersensitive reactions. This present study found that *α*-CD/MG inhibited the release of TNF-*α* in a concentration-dependent manner. The pro-inflammatory cytokine, TNF-*α*, critically mediates allergic and inflammatory responses. It can also increase leukocyte infiltration, T cell and neutrophil chemotaxis, and inflammation [41].

AF-343, a mixture of natural plant extracts from *Cassia tora* L., *Ulmus pumila* L., and *Taraxacum officinale-*fermented *Arctium lappa* fruit (F-AFE), and *licarin* A [40, 42, 43], significantly decreased the amount of TNF-*α* protein released and weakened the allergic impact that is triggered by IgE sensitisation. Similar to the *α*-CD/MG-mediated decrease in chronic and acute allergic characteristics, F-AFE has been found to decrease both chronic and acute inflammation by reducing the synthesis of inflammation mediators such as TNF-*α* from IgE-stimulated mast cells [42].

### 3.6. Suppression of IL-4 Release by *α*-CD/MG

**Figure 8:**
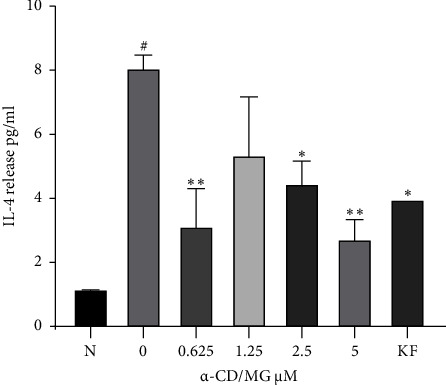
Effect of *α*-CD/MG on IL-4 release by activated RBL-2H3 cells. The RBL-2H3 cells were sensitised with IgE for 24 hours and then stimulated with 1 *μ*g/mL of DNP-BSA for 6 hours pretreatment with 0.625, 1.25, 2.5, and 5.0 *μ*M of *α*-CD/MG. An ELISA was used as per the manufacturer's instructions to measure the cytokine levels. The data of the two determinants (*n* = 2) are presented as mean ± SD, ^*∗*^*p* < 0.05, and ^*∗∗*^*p* < 0.001 indicating statistically significant in comparison with the untreated group. ^#^*p* < 0.05 in comparison with the normal group (N).

*α*-CD/MG at concentrations of 5.0, 2.5, and 0.625 *μ*M significantly inhibited the release of IL-4 compared to untreated cells (*p* < 0.001, 0.01, and 0.002, respectively). Furthermore, RBL-2H3 cells that were exposed to extended antigens have released inflammatory compounds, including pro-inflammatory cytokines, as a result of late-phase events [9].  Figure 8 shows that a 24-hour DNP-BSA challenge (5 *μ*Μ) significantly increased the amount of IL-4 released by IgE-antigen complex-stimulated cells. Nevertheless, the amount of *de novo* regulators produced by the IgE-antigen complex-stimulated RBL-2H3 cells significantly decreased when treated with 5, 2.5, and 0.625 *μ*Μ of *α*-CD/MG. Therefore, pretreatment with *α*-CD/MG significantly decreased the amount of *de novo* regulators produced better than IgE-antigen complex-stimulated cells.

Interleukin-4 (IL-4), an important pro-inflammatory cytokine, is released upon mast cell activation [14]. The present study showed that pretreatment with 5, 2.5, and 0.625 *μ*Μ of *α*-CD/MG significantly decreased the amount of IL-4 that IgE-antigen complex-stimulated RBL-2H3 cells produced. Fu et al. [14] similarly found that KF and coptisine decrease the overexpression of IL-4. More specifically, the use of 30, 20, or 10 *μ*M of coptisine led to fewer allergic events concerning *in vitro* IgE-mediated phenomena, including milder egg ovalbumin (OVA)-triggered allergic rhinitis in mice. Hence, researchers have highlighted the importance of using coptisine as an antiallergic substance [14].

Similar research has revealed a reduced release of IL-4 in different quantities when substances like KF, berberine, Shenqi, and other plant compounds were used [33, 44, 45]. Shenqi may exert an antiallergic impact by reducing mast cell degranulation and allergen synthesis due to the RBL-2H3 mast cells [44]. Immunoglobulin E/antigen (IgE/Ag) from the fisetin-based stimulation of RBL-2H3 cells significantly reduced IL-4 gene expression. Fisetin is a potent therapeutic substance for the IgE-regulation of allergic issues [46].

### 3.7. Effect of *α*-CD/MG on PGD2 Release

The results showed that *α*-CD/MG at concentrations of 5.0, 2.5, 1.25, and 0.625 *μ*M significantly inhibited the release of lipid mediator, PGD2, compared to the untreated cells (*p* < 0.001, 0.01, 0.002, and 0.001, respectively).

Mast cells synthesise PGD2, which is a critical aspect for the manifestation of allergic problems like asthma [47]. When RBL-2H3 cells are exposed to an antigen for extended periods, late-phase phenomena are created comprising the synthesis of *de novo* inflammatory regulators like the PGD2 lipid mediator. Hence, *α*-CD/MG exerts an antiallergic impact by reducing the synthesis of lipid mediators.

**Figure 9:**
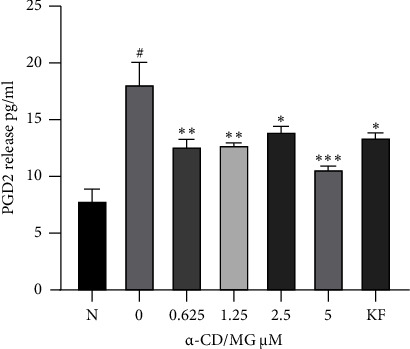
*α*-CD/MG suppressed PGD2 release by activated RBL-2H3 cells. The RBL-2H3 cells were sensitised with IgE for 24 hours and then stimulated with 1 *μ*g/mL of DNP-BSA for 6 hours pretreatment with 0.625, 1.25, 2.5, and 5.0 *μ*M of *α*-CD/MG. An ELISA was used as per the manufacturer's instructions to measure the cytokine levels. The data of the two determinants (*n* = 2) are presented as mean ± SD, ^*∗*^*p* < 0.05, ^*∗∗*^*p* < 0.001, and ^*∗∗∗*^p<0.0001 indicating statistically significant in comparison with the untreated group. ^#^*p* < 0.05 in comparison with the normal group (N).

Figure 9 demonstrates that DNP-BSA challenge for 24 hours led to a significant increase in the release of PGD2 in cells that were complex-stimulated with IgE-antigen. However, the presence of (5 *μ*Μ) *α*-CD/MG considerably reduced the levels of the aforementioned *de novo* mediators, which were released by the RBL-2H3 cells complex-stimulated with IgE-antigen. Compared to the IgE-antigen complex-stimulated cells, the release of *de novo* mediator was substantially reduced when pretreated with *α*-CD/MG.

The synthesis and release of the *de novo* lipid mediator, PGD2, was seen to be strongly reduced by *α*-CD/MG during the late-phase reaction in a concentration-dependent manner. This finding showed that *α*-CD/MG stabilised the distortion of the plasma membrane in the degranulating mast cells, which reduced the release of the prepared mediators. Moreover, *α*-CD/MG was also able to reduce the consequent release of the late-phase mediators into the extracellular environment. To accomplish this, it most likely targeted the signalling pathways that were responsible for producing these mediators [13, 44].

The results of this study indicated that the release of PGD2 from the mast cells was considerably reduced by *α*-CD/MG. A previous study similarly showed that there was a substantial decrease in the release of PGD2 from DNP-HSA-stimulated cells (50%) due to treatment with the phlorotannins, *Larix sbirica* extract (LSE), and 2,4,6-trihydroxy-3-geranylacetophenone (tHGA) at 20 *μ*M. During the late-phase reaction, tHGA significantly inhibited the production and release of PGD2 [9, 41, 48]. In RBL-2H3 cells stimulated by DNP-HSA, the inhibition of PGD2 through phlorotannins may prevent allergic reactions (e.g., hyperpermeability and bronchoconstriction) [48]. Accordingly, there is also a possibility that *α*-CD/MG can elicit an antiallergic activity in a similar manner. Moreover, the NF-kB and MAPK pathways are required for the mast cell production of PGD2 [41]. When the IgE mast cells were sensitised with the lipid mediator, PGD2, its production was considerably reduced by LSE and its active ingredients [41].

Moreover, an examination of the *in vivo* and *in vitro* effects of kaempferol indicated that there was a significant reduction in the production of PGD2. There was a decrease in the synthesis of PGD2 following the exposure of RBL-2H3 cells to DNP-BSA, and the treatment with 20 *μ*M of kaempferol. Alternatively, when mice were given 20 mg/kg of kaempferol after inhaling BSA, their lungs and PGD2 serum levels were suppressed [49]. Resveratrol was also significantly inhibited the production of PGD2 in skin mast cells when tested with a wide range of concentrations [47]. Previous studies have stated that there is a possibility that plant compounds such as tHGA, LSE, and kaempferol have the potential for therapeutic and/or prophylactic effects on allergic ailments caused by the activation of mast cells [9, 41, 49].

## 4. Conclusions

This research investigated the antiallergic properties of *α*-CD/MG on RBL-2H3 cells. The ^1^H-NMR analysis of *α*-CD/MG revealed shifts in the chemical peaks corresponding to the H7, H8, and H9 protons of MG, suggesting an interaction with *α*-CD. Additionally, TOCSY spectra displayed cross-correlation between the protons of MG and *α*-CD involved in the complex formation, particularly involving the H7, H8, and H9 protons of MG (benzyl moiety) and the H3 of *α*-CD. The findings demonstrated that *α*-CD/MG lowers the levels of pro-inflammatory and degranulation cytokines by inhibiting the release of histamine, IL-4, *β*-hexosaminidase, PGD2, and TNF-*α*. Moreover, *α*-CD/MG exhibited a notable reduction in mast cell degranulation and the release of both preformed and de novo mediators triggered by the IgE-antigen complex. The study suggested that *α*-CD/MG may be helpful in the treatment and prevention of allergic disorders by modulating the early and late stages of allergic reactions.

## Data Availability

The datasets used and analysed during the current study are available from the corresponding author on reasonable request.
